# HCV, HIV AND HBV rapid test diagnosis in non-clinical outreach settings can be as accurate as conventional laboratory tests

**DOI:** 10.1038/s41598-023-33925-2

**Published:** 2023-05-09

**Authors:** Milagros Muñoz-Chimeno, Jorge Valencia, Alvaro Rodriguez-Recio, Guillermo Cuevas, Alejandra Garcia-Lugo, Samuel Manzano, Vanessa Rodriguez-Paredes, Beatriz Fernandez, Lucía Morago, Concepción Casado, Ana Avellón, Pablo Ryan

**Affiliations:** 1 Hepatitis Unit, National Center of Microbiology, Carlos III Institute of Health, Madrid, Spain; 2 Infanta Leonor Hospital, Madrid, Spain; 3 Molecular Virology Unit, National Center of Microbiology, Carlos III Institute of Health, Madrid, Spain; 4 CIBERESP Epidemiology and Public Health, Madrid, Spain; 5 CIBER Infectious Diseases (CB 21/13/00044), Madrid, Spain; 6 Complutense University of Madrid, Madrid, Spain

**Keywords:** Infectious-disease diagnostics, Hepatitis, Hepatitis B virus, Hepatitis C virus, Virology, Retrovirus

## Abstract

Point of care rapid diagnostic tests (POC-RDT) for Hepatitis C virus (HCV), Human Immunodeficiency virus (HIV) and Hepatitis B virus (HBV), are ideal for screening in non-clinical outreach settings as they can provide immediate results and facilitate diagnosis, allowing high risk population screening. The aim of this study was to compare POC-RDT with laboratory conventional tests. A total of 301 vulnerable evaluable subjects (drug users, migrants and homeless population) were recruited at a mobile screening unit in outreach settings in Madrid. Fingerprick whole blood capillary samples were tested using the SD BIOLINE HCV POC-RDT, Determine HIV Early Detect and Determine HBsAg 2, and the results were assessed against the LIAISON XL HCV, HIV and Murex-HBsAg-Quant, reference assays, respectively. The feasibility and user satisfaction of the POC-RDT were evaluated through a questionnaire. The resolved sensitivity and resolved specificity and their 95% confidence intervals (95% CI) were as follows, respectively: SD-BIOLINE-HCV: 98.8% (95% CI 93.4, 100.0) and 100.0% (95% CI 98.3, 100.0); Determine HIV Early Detect: 100% (95% CI 85.2, 100.0) and 100% (95% CI 98.7, 100); and Determine HBsAg 2: 66.7% (95% CI 9.4, 99.2) and 100.0% (95% CI 98.7, 100.0). As expected, the number of subjects with a confirmed positive result for HBsAg was very low (n = 4). Therefore, the analytical sensitivity has been evaluated in addition: The Determine HBsAg 2 test demonstrated 100% sensitivity for standard concentrations ≥ 0.125 IU/mL. The subject questionnaire yielded positive feedback for most subjects. The POC-RDT fingerprick blood collection method was well received, and the tests demonstrated a comparable clinical performance with conventional tests in outreach settings and vulnerable high-risk populations.

## Introduction

It is estimated that globally there are 58 million people infected with Hepatitis C virus (HCV)^1^, 38 million persons worldwide are living with human immunodeficiency virus (HIV)^2^ and 296 million people who are chronically infected with Hepatitis B virus (HBV)^3^. HCV, HIV and HBV, are blood borne viruses that cause notifiable diseases, which consume health resources and have public health implications.

In recent years, taking advantage of the advent of new effective and safe antiviral treatments, the use of new diagnostic techniques and the implementation of innovative prevention strategies, the World Health Organization (WHO) has set targets and recommendations for the elimination and control of HCV^4,5^, HIV^6^ and HBV^7^ infections worldwide. Among other measures, these recommendations prioritize screening vulnerable populations at high risk for these infections and who have poor access to treatment.

Anti-HCV and anti-HIV total antibodies are considered the standard screening test for HCV and HIV infection^4^. Chronic HCV infection should be confirmed with RNA detection^8,9^ while HIV antibody reactivity should be confirmed by an additional two serology reactive test results for a HIV-positive diagnosis^10^. HBsAg is the earliest indicator (besides HBV DNA detection) of HBV acute or chronic infection, may be present before symptoms appear, and can thus be used to detect active infection^11^. Point of care rapid diagnostic tests (POC-RDT) can provide results within around 30 min with a minimal need for additional supplies or instrumentation and could be ideal for screening and monitoring purposes in HCV, HIV and HBV, infections in outreach or resource limited settings. The SD BIOLINE HCV (now Bioline HCV), the Determine HIV Early Detect and the Determine HBsAg 2 tests are rapid antibody and antigen detection lateral flow tests that are simple to use, require a small amount of whole blood and produce results within minutes.

The aim of this study was to compare the sensitivity, specificity and accuracy of the mentioned POC-RDT versus conventional laboratory assays for the diagnosis of HCV, HIV and HBV in outreach settings. In the case of HBsAg, a panel was used to determine the analytical sensitivity, being the panel a dilution series of the WHO international reference preparation. Additionally, the patient perception of the procedure was evaluated.

## Materials and methods

### Study population and design

We carried out a prospective study in Madrid, Spain, from June 2019 to August 2019. We approached and conducted screening for HCV, HIV and HBV in subjects with a high risk for acquiring blood borne infections in Madrid’s hot spots, namely, mobile harm reduction units, and institutions providing social assistance, public areas, homeless shelters, and places where street prostitution is practiced. The study was conducted using a screening mobile van (https://unidadmovil.es/) that travelled to outreach settings where these vulnerable populations gathered. Subjects were selected consecutively in the order of appearance. We included subjects who fulfilled the following criteria: (1) aged ≥ 18 years; (2) faced discrimination because of social, health, economic, and cultural issues; and (3) were capable of signing the informed consent form.

### Ethics statement

The study was conducted according to the Declaration of Helsinki, and before any study-related procedures were performed, each subject was required to read, sign, and date the ethics committee-approved informed consent form explaining the nature, purpose, risks, and duration of the study. The study and informed consent forms were approved by the Institutional Review Board and the Research Ethics Committees, the Instituto de Salud Carlos III Ethics Committee (report CEI PI 25_2019-v3) and the Hospital General Universitario Gregorio Marañón Ethics Committee (378/18 and MP-001/2019).

### Data sources

Epidemiological data (age, sex, nationality) and the history of HIV and/or hepatitis diagnosis and treatment were collected. Anonymized study data were entered into an electronic data capture system provided by the study sponsor. Further information on substance abuse (daily alcohol intake, benzodiazepine use, and illegal drug use) and homeless status was collected by the investigators through a questionnaire on a mobile device with internet connection. Data was stored using the Research Electronic Data Capture system (REDCap, Vanderbilt University, Nashville, TN, USA) which is hosted at the Ideas for Health Association.

### Sample size

At least 300 evaluable subjects were intended to be enrolled in the study. The number of HCV, HIV and HBsAg positive case findings, and the specificity of the tests, were to be reported. The number of negative samples was determined as follows: The number of negative HCV tests required was 164 based on 99% specificity with low-limit = 96% at one-sided 97.5% CI. The simultaneous goal was to achieve 80% power for point estimate Sp ≥ 99% with 95% confidence (one-sided) lower bound > 97%, assuming Sp = 99.6%. A minimum sample size of N = 156 HIV negative achieves 87% power. The required HBsAg negative number was 204 based on 98% specificity with low-limit = 95% at one-sided 97.5% 95% CI. Therefore, the required minimum negative sample size for HCV, HIV and HBV was 204. It was expected that 9–30 subjects would be HBsAg-positive and that 20–70 subjects would be HCV antibody-positive^12–16^. As the number of positive HBV was expected to be low, a complementary HBV study was carried out.

### General workflow of the study (Fig. 1)

**Figure 1:**
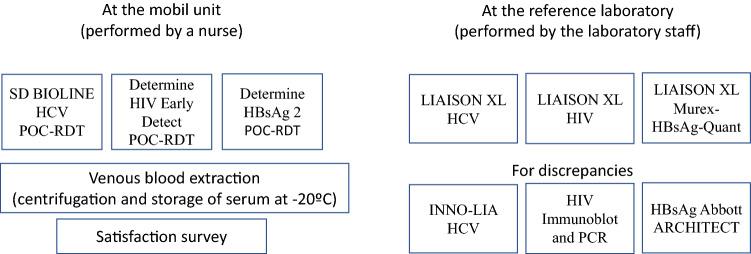
Graphic workflow of the study.

Potential participants were approached with the mobile unit and offered to participate by the social workers. Once they agree and sign informative consent, a nurse perform and read the POC-RDT. Venous blood is then obtained by the nurse, and sera were separated, sent to the reference laboratory and stored at – 80 °C.

### Point of care rapid diagnostic testing

After informed consent was obtained, fingerprick capillary whole blood specimens were collected by a nurse from each subject using lancets (ACCU-CHEK: Accu-Chek Safe-T Pro Plus, Roche Diagnostics) EDTA capillary blood (CB) collection tubes MICROSAFE capillary blood collection tube, which where appropriate for the RDT of the study. The fingerprick capillary samples were mostly obtained from a single finger puncture but additional punctures could be performed if needed. To reduce the risk of specimen contamination during fingerprick collection, proper cleansing procedures were followed, and the first droplet of blood was wiped off with sterile gauze or a cotton ball. The whole blood specimens were immediately tested as follows: 10 μL of CB for HCV antibody, 50 μL of CB for HIV testing and 50 µL of CB for HBsAg. For this study, we used the SD BIOLINE HCV test (Abbott Diagnostics, USA, Ref 02FK10CE, 02FK16CE, 02FK17CE) for HCV; the Determine HIV Early Detect (Abbott Diagnostics, USA, Ref 7D2846, 7D2847)) (previously named “Alere™ HIV Combo”) test for HIV; and the Determine HBsAg 2 test (Abbott Diagnostics, USA, Ref 7D2946, 7D2947) for HBV. The SD BIOLINE HCV test is designed for the qualitative detection of antibodies specific to HCV (targeting Core, NS3, NS4, NS5 antigens) in human serum, plasma, venous whole blood or finger prick whole blood^17^. The Determine™ HIV Early Detect test is an in vitro, visually read, qualitative, lateral-flow immunoassay for the detection of antibodies (Ab) against HIV-1 and HIV-2 in one line and the detection of non-immuno-complexed HIV-1 p24 antigen (Ag) in a separate line in human capillary and venous whole blood, plasma and serum^18^. The Determine HBsAg 2 is an in vitro, visually read, qualitative immunoassay for the detection of HBsAg in human serum, plasma or whole blood^19^. Each rapid test was performed once according to the manufacturer’s instructions for use. An invalid test could be repeated if the patient provided consent. Testing was performed by a nurse who also read the test and was blinded to the subjects’ clinical HCV, HIV and HBV status. For all tests, a diluent/chase buffer (specific diluent included in each kit, for each test) was added immediately after sample application to the test device. The tests were interpreted at the following times after diluent/chase buffer application: 5–20 min for HCV, 20–40 min for HIV and 15–30 min for HBsAg. Any reads performed outside this time window were considered invalid. Subjects were provided with their rapid test results. Those patients with reactive results were offered a confirmatory test according to the standard circuit of the mobile unit.

Manufacturer’s instructions for use are available at their web pages^17,18,20^.

### Subject questionnaire

A questionnaire was administered to the study subjects through the study staff, asking the following questions: (i) was the fingerprick painful? The response options were: no, acceptable, very painful; (ii) How painful was the fingerprick in comparison to venipuncture? The response options were: More painful than venepuncture, equally painful compared to venipuncture, less painful than the venepuncture. The number of fingerpicks required for the three tests were also recorded.

### Linkage to care

All subjects with a reactive test were offered referral to the hospital the same day because treatment for HCV, HIV or HBV in Spain can only be prescribed at hospitals by a specialist physician. Due to the geographic proximity and the established protocols for referral most of the patients were accompanied to the Fast-Track Clinic at “Infanta Leonor” Hospital.

### Reference testing

Venepuncture was performed utilizing the site’s standard blood collection method to collect a 10 mL sample that was centrifuged to obtain a serum specimen. Serum samples were aliquoted and frozen at − 80 °C on the day of sample collection and were used for batch-testing with HCV, HIV and HBsAg reference assays. All retained serum samples were stored at − 80 °C until the end of the study. Testing was performed by trained laboratory professionals in the reference laboratory who followed the standard operating procedures for sample collection, processing, and testing.

Reference tests were as follows: the indirect chemiluminescence assay LIAISON XL HCV (DiaSorin SpA, Italy) and immunoblot INNO-LIA HCV (Fujirebio, Japan) for HCV; the chemiluminescent immunoassay for the simultaneous qualitative detection of HIV p24 antigen and antibodies to HIV-1 (Groups M and O) and HIV-2 LIAISON® XL HIV (DiaSorin SpA, Italy) and HIV Immunoblot and in-house PCR for HIV; and the direct two-step sandwich chemiluminescence assay LIAISON XL Murex HBsAg Quant (DiaSorin SpA, Italy) and the chemiluminescent microparticle immunoassay (CMIA) HBsAg Abbott ARCHITECT (Abbott Diagnostics, USA) tests were used for HBV detection. Low reactive results with LIAISON XL Murex and negative HBsAg ARCHITECT results were considered as inconclusive, and were discarded. Two different cut-off values were considered for HBsAg: the analytical 0.05 IU/mL and the 0.13 IU/mL required by European regulations.

If a subject was found to have a discrepant venous sample result as compared to the POC-RDT result, the subject was contacted and requested for a retest.

### Complementary study for HBV

An evaluation of an HBsAg-negative pooled serum sample and an HBsAg WHO NIBSC 12/226 international standard was performed. The samples were diluted as follows: 1 IU/mL, 0.5 IU/mL, 0.25 IU/mL, 0.167 IU/mL, 0.125 IU/mL and 0.1 IU/mL. The negative pooled serum and dilution panel samples were tested using both the Determine™ HBsAg 2 and the reference technique with three replicates each, read by three blinded readers in a randomized fashion.

### Statistical analysis

Sensitivity was calculated as true positive (TP)/(TP + false negative (FN)). Specificity was calculated as true negative (TN)/TN + false positive (FP)). Accuracy was calculated as (TN + TP)/(TN + TP + FN + FP). Calculation of 95% confidence intervals was done by the Exact (Clopper-Pearson) method. All analyses were performed SAS 9.4 (SAS Inc., Cary, NC, USA). All p-values were two-tailed and *p* < 0.05 was considered statistically significant.

## Results

**Figure 2:**
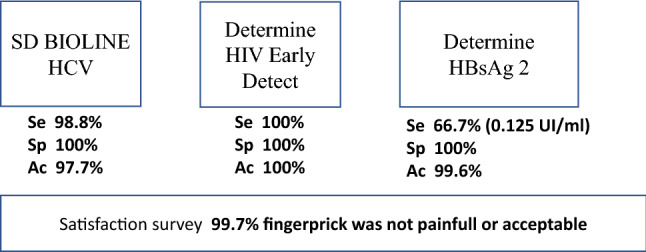
Graphic summary of the results. *Se* sensitivity, *Sp* specificity, *Ac* accuracy.

Of the 302 enrolled subjects, one was excluded from the study because this patient refused venepuncture for sample collection. The demographics and clinical characteristics of the 301 subjects are shown in Tables 1 and 2, respectively. Mean age was 45 (SD 11) years. Most study subjects (55.5%) were born in Spain with the remaining subjects originating from 36 other countries. Among the 110 female subjects, 109 subjects were not pregnant while the information was missing for 1 subject. In total, 76 subjects (25.2%) had a prior diagnosis of HCV, 22 subjects (7.3%) had an HIV diagnosis and 19 subjects (6.3%) had a past diagnosis of HBV. Existing coinfections and history of treatment according to the questionnaire are detailed in Table 2. Results of each test as follows are summarized in Fig. 2.

**Table 1:** Participant demographics.

		N	**%**
Age (Missing n = 2, 0.66%)	N	299	
	Mean	45	
	Standard Deviation	11.0	
	Median	45	
	Min–Max	19, 76	
Gender	Female	110	36.5%
	Male	191	63.5%
Origin	Outside Spain	134	44.5%
	Spain	167	55.5%

**Table 2:** Participant medical history according to participant answers in questionnaire.

	N	%
No history of HBV, HCV HBV	211	70.1%
HBV		
Past/current history of HBV	19	6.3%
HBV only	8	2.7%
HBV + HCV	9	3.0%
HBV + HCV + HIV	1	0.3%
HBV + HIV	1	0.3%
Current HBV with medication	3	1.0%
Current HBV without medication	16	5.3%
HCV		
Current HCV	76	25.2%
HCV	51	16.9%
HCV + HIV	15	5.0%
HBV + HCV	9	3.0%
HBV + HCV + HIV	1	0.3%
Current HCV with medication	24	8.0%
Current HCV without medication	52	17.3%
HIV		
Current HIV	22	7.3%
HIV	5	1.7%
HBV + HIV	1	0.3%
HCV + HIV	15	5.0%
HBV + HCV + HIV	1	0.3%
Current HIV with medication	22	7.3%
Current HIV without medication	0	0%
Past Hepatitis A	15	5.0%

### HCV results

HCV screening results are detailed in Table 3. Out of the 301 evaluable samples, 7 were excluded from further analysis because the results were read outside the test window of 5–20 min. Of the remaining 294 samples, 211 samples tested negative by both the SD BIOLINE HCV and the reference LIAISON XL HCV assays. A total of 81 samples tested reactive by both the SD BIOLINE HCV and LIAISON XL HCV assay, while one sample tested reactive by LIAISON XL + INNO-LIA and negative by SD BIOLINE. Hence it gave a sensitivity of 98.8% (95% CI 93.4, 100.0), a specificity of 100.0% (95% CI 98.3, 100.0), and an accuracy of 99.7% (95% CI 98.1, 100.0) for the SD BIOLINE HCV assay, as shown in Table 3.

**Table 3:** Sensitivity, specificity and accuracy of the SD BIOLINE HCV assay.

SD BIOLINE HCV	LIAISON XL HCV			LIAISON XL HCV with discrepant results resolved with INNO-LIA		
	Reactive	Negative	Total	Reactive	Negative	Total
Reactive	81^(2)^	0	81	81^(2)^	0	81
Negative	2^(1)^	211	213	1	212	213
Total	83	211	294	82	212	294
Sensitivity (95% CI) (%)			97.6 (91.6, 99.7)			98.8 (93.4, 100.0)
Specificity (95% CI) (%)			100.0 (98.3, 100.0)			100.0 (98.3, 100.0)
Accuracy (95% CI) (%)			99.3 (97.6, 99.9)			99.7 (98.1, 100.0)

^(1)^The INNO-LIA confirmation assay was positive for discrepant sample #1 and negative for discrepant sample#2.

^(2)^Ten patients without a previous HCV diagnosis had positive results with both SD BIOLINE and LIAISON XL HCV.

### HIV results

HIV Ag were negative in all assayed samples. HIV screening results for HIV Ab are detailed in Table 4. Out of the 301 assessable samples, 6 were excluded from further analysis because the results were read outside the test window of 20–40 min (N = 5), or the HIV test was invalid and was not repeated (N = 1). Of the remaining 295 samples, 272 samples tested Ab negative by both the Determine HIV Early Detect test and the reference LIAISON XL HIV + Inmmunoblot assay. A total of 23 samples tested Ab reactive by both the Determine HIV Early Detect test and the reference LIAISON XL HIV assay. Then it gave a sensitivity of 100% (95% CI 85.2, 100.0), a specificity of 100% (95% CI 98.7, 100) and an accuracy of 100% (95% CI 98.8, 100) for the Determine HIV Early Detect test, as shown in Table 4.

**Table 4:** Sensitivity, specificity and accuracy of the determine HIV early detect test in 295 evaluable samples.

Determine HIV Early Detect (antibodies-Ab)	LIAISON XL HIV (antibodies-Ab)			LIAISON XL HIV with discrepant results resolved with Immunoblot and PCR		
	Reactive	Negative	Total	Reactive	Negative	Total
Reactive	23^(1)^	0	23	23^(1)^	0	23
Negative	1	271	272	0	272	272
Total	24	271	295	23	272	295
Sensitivity (95% CI) (%)			95.8 (78.9, 99.9)			100.0 (85.2, 100.0)
Specificity (95% CI) (%)			100.0 (98.6, 100.0)			100.0 (98.7, 100.0)
Accuracy (95% CI) (%)			99.7 (98.1, 100.0)			100.0 (98.8, 100.0)

^(1)^One patient without a previous HIV diagnosis had positive results on both Determine HIV Early Detect and LIAISON XL HIV.

### HBV results

HBV screening results are detailed in Table 5. Out of the 301 evaluable samples, 5 were excluded from further analysis because the results were read outside the test window of 15–30 min. Of the remaining 296 samples, 19 were excluded from the analysis because results were considered inconclusive (since they had low positive result with LIAISON XL and were HBsAg ARCHITECT negative). Among the remaining 277 samples, a total of 273 tested negative by both the Determine HBsAg 2 and reference assays. Two samples were reactive by the reference assays and by the Determine HBsAg 2 assay. Finally, another two samples were reactive by the reference assays (Sample 1: 0.18 IU/mL (LIAISON XL) and 0.54 IU/mL (ARCHITECT); Sample 2: 0.08 IU/mL (LIAISON XL) and 0.081 IU/mL (ARCHITECT)) and negative by the Determine HBsAg 2 assay). The Determine HBsAg 2 sensitivity, specificity and accuracy were then determined as 50.0% (95% CI 6.8, 99.2), 100.0% (95% CI 98.7, 100.0) and 99.3% (95% CI 97.4, 99.9), respectively. Using the cut-off 0.13 IU/mL, as required by European regulations, Determine HBsAg 2 gave a sensitivity, specificity and accuracy of 66.7% (95% CI 9.4, 99.2), 100.0% (95% CI 98.7, 100.0) and 99.6% (95% CI 98.1, 100.0), respectively, as shown in Table 5. An additional analytical sensitivity evaluation of Determine HBsAg 2 using a serum dilution panel was carried out. The Determine HBsAg 2 test was able to detect 100% of HBsAg samples (all three replicates) at concentrations of ≥ 0.125 IU/mL, and 1 of 3 replicates at 0.1 IU/mL.

**Table 5:** Sensitivity, specificity and accuracy of the determine HBsAg 2 assay.

Determine HBsAg 2	LIAISON XL Murex HBsAg Quant combined with Abbott ARCHITECT cut-off = 0.05 IU/mL			LIAISON XL Murex HBsAg Quant combined with Abbott ARCHITECT, cut-off = 0.13 IU/mL		
	Reactive	Negative	Total	Reactive	Negative	Total
Reactive	2	0	2	2	0	2
Negative	2^(1)^	273	275	1	274	275
Total	4	273	277	3	274	277
Sensitivity (95% CI) (%)			50.0 (6.8, 93.2)			66.7 (9.4, 99.2)
Specificity (95% CI) (%)			100.0 (98.7, 100.0)			100.0 (98.7, 100.0)
Accuracy (95% CI) (%)			99.3 (97.4, 99.9)			99.6 (98.0, 100.0)

^(1)^Discrepant Sample #1: LIAISON XL: 0.18 IU/mL and ARCHITECT: 0.54 IU/mL; Discrepant Sample #2: LIAISON XL: 0.081 IU/mL and ARCHITECT: 0.080 IU/mL.

### Satisfaction survey

Finally, regarding results of the questionnaire, a total of 255 subjects (84.7%) responded that the fingerprick was not painful; 45 subjects (15.0%) responded that the pain of the fingerprick was acceptable, and one subject (0.3%) responded that the fingerprick was very painful. Comparing fingerprick with venipuncture, 184 subjects (61.1%) considered the fingerprick to be equally painful to venipuncture, 95 subjects (31.6%) considered it to be less painful than venipuncture, and 22 subjects (7.3%) considered the fingerprick to be more painful than venipuncture. Regarding the number of needed fingerpricks for the three tests, 293 subjects (97.3%) required only a single fingerprick, 7 subjects (2.3%) required 2 fingerpricks, and 1 subject (0.3%) required 4 fingerpricks to obtain a sufficient amount of blood for testing.

## Discussion

In this study we evaluated the sensitivity, specificity and accuracy of three HCV, HIV and HBV POC-RDT in comparison with gold standard conventional tests performed in a laboratory. The participants of the study were selected at a screening mobile unit in Madrid, which offers screening and linkage to care to outreach and vulnerable populations. Blood samples were drawn at the mobile unit after informed consent was signed. From the questionnaires, it could be concluded that the acceptance of the rapid tests in this population was good. The study uptake was excellent among those approached and the fingerprick procedure was well received.

The number of recruited positive and negative patients for HCV and HIV testing in the target population was similar to the number that was expected. Compared to the gold standard for HCV diagnosis, LIAISON XL HCV with discrepant result resolution using INNO-LIA, the SD BIOLINE rapid test achieved optimal resolved sensitivity, resolved specificity and resolved accuracy (98.8%, 100% and 99.7% respectively). One unique patient was negative by the rapid test and positive by both HCV antibody reference tests had a medical history of HCV infection and was negative for HCV RNA, suggesting a presence of residual antibodies in a non-active HCV infection. A total of 10 patients who tested positive were unaware of their diagnosis (3.3%) and 4 of these had an active HCV infection (data not shown). Among the tested, 22 of those with a positive rapid test, had received a prior HCV diagnosis but were had not been treated with antivirals and were consequently referred to the HCV clinic for care. For HIV diagnosis, the Determine HIV Early Detect test had a resolved sensitivity of 100%, a resolved specificity of 100% and a resolved accuracy of 100%, compared to the LIAISON XL HIV with discrepant result resolved using Immunoblot and PCR. One subject without a prior HIV diagnosis was diagnosed with HIV. Performance parameters of tests agree with those provided by the manufacturers (links provided in material and methods).

In contrast, only 4 recruited subjects were HBsAg-positive (3 using the 0.13 IU/mL cut-off) and, therefore, represented a small sample size that was a limitation for the Determine HBsAg 2 test sensitivity calculation in the screening study. Recent prevalence results in the Spanish general population published during the recruitment period indicated a HBsAg prevalence of 0.19%^21^, which was in agreement with the low number of HBV-positive subjects. Although the clinical sensitivity of the test was previously calculated using a larger panel of positives and also comparing RBT with conventional test, and reached 97.2% for fingerprick samples^19^, an additional evaluation was performed to assess the analytical sensitivity in the current study. After testing serial dilutions of the HBsAg WHO standard, a limit of detection of 0.125 IU/mL was established, which is under the 0.13 IU/mL limit required by European regulations. The specificity and accuracy of the Determine HBsAg 2 test were 100% and 99.6% respectively. Two subjects with a past diagnosis of HBV who were not taking medication, were identified as HBsAg current chronic carriers. Consequently, in view of the good analytical sensitivity results and the previous calculation of clinical sensitivity^19^, we can conclude that although the limitation of the low number of positive participants, the Determine HBsAg 2 test would also be as accurate as conventional test.

In this study we have evaluated three POC-RDT in the setting where they are most needed (outside a health centre or laboratory) and we have tested a vulnerable target population providing treatment to patients who were positive for HCV, HIV or HBV. The WHO plan for the elimination and control of viral hepatitis and HIV, recommends improving access to the healthcare system in high-risk populations. The use of POC-RDT is an important strategy, not only to diagnose and treat these populations, but also to monitor the prevalence of these diseases at the community level. Guidelines recommend that access to HCV, HIV or HBV testing be expanded and routinely offered in non-specialist settings in order to reach out to high-risk and marginalised sections of the population and reduce late presentations^22,23^. To maximize the benefits of implementation of POC-RDT, health care providers require appropriate training and supervision to offer and administer POC-RDT^24^.

Systematic reviews and meta-analysis to evaluate the diagnostic accuracy of available rapid diagnostic tests in detecting antibodies to HCV have been published showing a high overall sensitivity and specificity compared to laboratory-based EIAs^25^. With the increasing availability of effective treatments for HCV, HIV and HBV, countries are seeking testing kits with high sensitivity and specificity, to allow them to scale up screening, especially among at-risk populations^26^. Performance, cost, and accessibility need to be considered. Other meta-analyses have shown that many people living with HIV (PLHIV) are HBV and/or HCV co-infected, and that the triple infection of HCV, HIV and HBV causes more clinically unfavourable consequences than mono- or dual infections^27,28^. Furthermore, these studies have shown that people who inject drugs (PWID) are severely and disproportionately affected by HCV, HIV and HBV, and that the prevalence of HIV/HCV coinfection is highest among PWID as compared with other high-risk groups and the general population^27,29^. In some regions such as sub-Saharan Africa and south Asia, HIV prevalence is higher among women, and is also higher among women who inject drugs in some countries^30^. These studies highlight the urgent need for HCV, HIV and HBV testing and outreach, especially among groups considered to be at high risk such as PWID and among more vulnerable groups such as women.

The ease of use and immediately available results provide significant advantages for point-of-care testing in cohorts that are hard to reach, such as PWID, homeless people and migrants. The mobile unit set up for the screening program could easily accommodate the point-of-care testing. Testing in this setting allows diagnosis and treatment of individuals who would otherwise not receive a diagnosis and be subsequently would not be referred to appropriate treatment pathways^31^. The SD BIOLINE HCV, the Determine HIV Early and the Determine HBsAg 2 Detect tests, have good sensitivity, specificity and accuracy compared to the reference assays. Results are produced within minutes and the fingerprick blood collection procedure was well received by the subjects. These rapid diagnostic tests are ideal for use in non-clinical outreach settings for populations who might otherwise be unlikely to have access to diagnostic HCV, HIV and HBV, testing.

## Supplementary Information

Supplementary Information.

## Data Availability

Raw data generated during this study are included in this article as Supplementary Material.
