# Supplementary material for: HCV, HIV AND HBV rapid test diagnosis in non-clinical outreach settings can be as accurate as conventional laboratory tests
Source: Sci Rep. 2023 May 9;13:7554. doi: 10.1038/s41598-023-33925-2 (PMC10170094; doi:10.1038/s41598-023-33925-2)
Supplement: Supplementary file 1 — Supplementary Information. [file 41598_2023_33925_MOESM1_ESM.pdf]

Supplementary material: raw data and variable descriptions

site	subjID	age	gender	spain	pregyn	eligible	diag	Hep_A	Hep_B	Hep_C	HIV	Hep_A_M	Hep_B_M	Hep_C_M	HIV_M	HBV_F	td_HBV	HBV_OTW	HBV_S1	HBV_S2	HCV_F	td_HCV	HCV_OTW	HIV_F	td_HIV	HIV_OTW	pcrname	PCR	liais_hbs	neutryn	antihbc	abbarch	liais_hcv	innolia	liais_hiv	immblot	painfs	painfsven	manyfs
01	01-0001	24	M	0		Yes	NON	NA	NA	NA	NA	NA	NA	NA	NA	NEG	17		NEG	NEG	NEG	6		NEG	25		HBV	NEG	0,056		NEG	<0,05	NEG		NEG		NOT	EQUAL	2
01	01-0002	54	F	1	N	Yes	NON	P	NA	NA	NA	N	NA	NA	NA	NEG	15				NEG	6		NEG	22				<0,03				NEG		NEG		NOT	EQUAL	1
01	01-0003	59	M	1		Yes	NON	NA	NA	NA	NA	NA	NA	NA	NA	NEG	17				NEG	6		NEG	24				NR-0,035				NEG		NEG		NOT	EQUAL	1
01	01-0004			0		No																																	
01	01-0005	45	M	1		Yes	NON	NA	NA	NA	NA	NA	NA	NA	NA	NEG	17				NEG	5		NEG	28				<0,03				NEG		NEG		NOT	EQUAL	1
01	01-0006	55	M	0		Yes	HCV	NA	NA	C	NA	NA	NA	N	NA	NEG	24				POS	6		NEG	34		HCV	NEG	<0,03				POS		NEG		NOT	MORE	1
01	01-0007	38	M	1		Yes	NON	NA	NA	NA	NA	NA	NA	NA	NA	NEG	21				NEG	7		NEG	31				<0,03				NEG		NEG		NOT	EQUAL	1
01	01-0008	46	F	0	N	Yes	NON	P	NA	NA	NA	N	NA	NA	NA	NEG	19		NEG	NEG	NEG	6		NEG	32		HBV	NEG	0,051		POS	<0,05	NEG		NEG		NOT	EQUAL	1
01	01-0009	24	F	1		Yes	NON	NA	NA	NA	NA	NA	NA	NA	NA	NEG	16				NEG	8		NEG	25				NR-0,042				NEG		NEG		NOT	EQUAL	1
01	01-0010	40	M	1		Yes	NON	NA	NA	NA	NA	NA	NA	NA	NA	NEG	15				NEG	6		NEG	24				<0,03				NEG		NEG		NOT	EQUAL	1
01	01-0011	53	M	1		Yes	NON	NA	NA	NA	NA	NA	NA	NA	NA	NEG	20				NEG	6		NEG	26				<0,03				NEG		NEG		NOT	EQUAL	1
01	01-0012	63	F	1	N	Yes	NON	NA	NA	NA	NA	NA	NA	NA	NA	NEG	17		NEG		NEG	9		NEG	24		HBV	NEG	0,058		POS	<0,05	NEG		NEG		NOT	EQUAL	1
01	01-0013	52	F	1	N	Yes	HCV_HIV	NA	NA	C	C	NA	NA	Y	Y	NEG	17				POS	7		POS	23		HCV	NEG	<0,03				POS		POS		NOT	EQUAL	1
01	01-0014	35	M	1		Yes	NON	NA	NA	NA	NA	NA	NA	NA	NA	NEG	16				NEG	6		NEG	25				<0,03				NEG		NEG		ACCEPT	MORE	1
01	01-0015	60	M	1		Yes	NON	NA	NA	NA	NA	NA	NA	NA	NA	NEG	16				NEG	10		NEG	22				<0,03				NEG		NEG		NOT	EQUAL	1
01	01-0016	32	F	1	N	Yes	NON	NA	NA	NA	NA	NA	NA	NA	NA	NEG	17				NEG	7		NEG	29				<0,03				NEG		NEG		NOT	EQUAL	1
01	01-0017	30	M	1		Yes	NON	NA	NA	NA	NA	NA	NA	NA	NA	NEG	16				NEG	7		NEG	24				<0,03				NEG		NEG		NOT	EQUAL	1
01	01-0018	42	M	1		Yes	NON	NA	NA	NA	NA	NA	NA	NA	NA	NEG	17				NEG	7		NEG	26				NR-0,043				NEG		NEG		NOT	EQUAL	1
01	01-0019	57	M	1		Yes	HBV	NA	P	NA	NA	NA	N	NA	NA	NEG	18				NEG	6		NEG	30				<0,03				NEG		NEG		NOT	EQUAL	1
01	01-0020	42	F	1	N	Yes	NON	NA	NA	NA	NA	NA	NA	NA	NA	NEG	17				NEG	6		NEG	26				<0,03				NEG		NEG		NOT	MORE	1
01	01-0021	41	M	1		Yes	NON	NA	NA	NA	NA	NA	NA	NA	NA	NEG	16				NEG	6		NEG	23				<0,03				NEG		NEG		NOT	EQUAL	1
01	01-0022	50	M	1		Yes	HCV	NA	NA	C	NA	NA	NA	N	NA	NEG	17				NEG	5		NEG	24				NR-0,04				NEG		NEG		NOT	LESS	1
01	01-0023	60	M	1		Yes	HBV	NA	C	NA	NA	NA	N	NA	NA	NEG	16				NEG	7		NEG	22				<0,03				NEG		NEG		NOT	LESS	1
01	01-0024	34	F	1	N	Yes	HCV	NA	NA	C	NA	NA	NA	N	NA	NEG	17				POS	6		NEG	26		HCV	NEG	<0,03				POS		NEG		NOT	LESS	1
01	01-0025	41	M	0		Yes	NON	NA	NA	NA	NA	NA	NA	NA	NA	NEG	17				NEG	5		NEG	25				<0,03				NEG		NEG		NOT	EQUAL	1
01	01-0026	66	M	1		Yes	HCV	NA	NA	C	NA	NA	NA	N	NA	NEG	17				POS	6		NEG	25		HCV	POS	<0,03				POS		NEG		NOT	EQUAL	2
01	01-0027	50	M	1		Yes	HCV	NA	NA	C	NA	NA	NA	N	NA	NEG	18				POS	7		NEG	22		HCV	NEG	<0,03				POS		NEG		NOT	EQUAL	1
01	01-0028	28	M	1		Yes	NON	NA	NA	NA	NA	NA	NA	NA	NA	NEG	20				NEG	8		NEG	21				<0,03				NEG		NEG				
01	01-0029	49	M	1		Yes	NON	NA	NA	NA	NA	NA	NA	NA	NA	NEG	17				NEG	6		NEG	23				<0,03				NEG		NEG		NOT	EQUAL	1
01	01-0030	51	M	1		Yes	HBV_HCV	NA	C	C	NA	NA	N	Y	NA	NEG	17				POS	8		NEG	24		HCV	NEG	<0,03				POS		NEG		NOT	EQUAL	1
01	01-0031	36	M	1		Yes	NON	NA	NA	NA	NA	NA	NA	NA	NA	NEG	17				NEG	7		NEG	26				<0,03				NEG		NEG		NOT	EQUAL	1
01	01-0032	57	M	1		Yes	HBV	NA	C	NA	NA	NA	N	NA	NA	NEG	17				NEG	6		NEG	25				<0,03				NEG		NEG		VERY	LESS	1
01	01-0033	62	F	1	N	Yes	HBV	NA	C	NA	NA	NA	N	NA	NA	NEG	16				NEG	6		NEG	24				<0,03				NEG		NEG		ACCEPT	LESS	1
01	01-0034	55	M	1		Yes	HBV_HCV	NA	C	C	NA	NA	N	N	NA	NEG	17				POS	5		NEG	24		HCV	NEG	<0,03				POS		NEG		NOT	EQUAL	1

01	01-0035	46	M	0		Yes	NON	NA	NA	NA	NA	NA	NA	NA	NA	NEG	17			NEG	7	NEG	25			<0,03			NEG	NEG	NOT	EQUAL	1
01	01-0036	56	M	0		Yes	NON	NA	NA	NA	NA	NA	NA	NA	NA	NEG	19			NEG	7	NEG	26			<0,03			NEG	NEG	NOT	EQUAL	1
01	01-0037	44	M	1		Yes	HCV	NA	NA	C	NA	NA	NA	N	NA	NEG	17			POS	7	NEG	24	HCV	POS	<0,03			POS	NEG	NOT	EQUAL	1
01	01-0038	57	M	0		Yes	HCV_ HIV	NA	NA	C	C	NA	NA	N	Y	NEG	17			POS	6	POS	24	HCV	NEG	<0,03			POS	POS	NOT	LESS	4
01	01-0039	45	F	1	N	Yes	HCV	NA	NA	C	NA	NA	NA	N	NA	NEG	18			POS	6	NEG	23	HCV	NEG	<0,03			POS	NEG	ACCEPT	EQUAL	1
01	01-0040	19	M	0		Yes	NON	NA	NA	NA	NA	NA	NA	NA	NA	NEG	19			NEG	7	NEG	23			<0,03			NEG	NEG	NOT	LESS	1
01	01-0041	23	M	1		Yes	NON	NA	NA	NA	NA	NA	NA	NA	NA	NEG	18			NEG	6	NEG	23			<0,03			NEG	NEG	NOT	LESS	1
01	01-0042	50	M	1		Yes	HIV	NA	NA	NA	C	NA	NA	NA	Y	NEG	8	1		NEG	5	POS	22			<0,03			NEG	POS	NOT	EQUAL	2
01	01-0043	49	M	1		Yes	HBV_ HCV	NA	C	C	NA	NA	N	N	NA	NEG	15			POS	6	NEG	23	HCV	POS	<0,03			POS	NEG	NOT	EQUAL	1
01	01-0044	40	M	1		Yes	NON	NA	NA	NA	NA	NA	NA	NA	NA	NEG	17			NEG	6	NEG	24			<0,03			NEG	NEG	NOT	LESS	1
01	01-0045	37	F	1	N	Yes	NON	NA	NA	NA	NA	NA	NA	NA	NA	NEG	18			NEG	6	NEG	25			<0,03			NEG	NEG	NOT	EQUAL	1
01	01-0046	45	M	0		Yes	NON	NA	NA	NA	NA	NA	NA	NA	NA	NEG	19			NEG	12	NEG	29			<0,03			NEG	NEG	NOT	EQUAL	1
01	01-0047	51	M	1		Yes	HCV	NA	NA	C	NA	NA	NA	Y	NA	NEG	18			POS	5	NEG	24	HCV	NEG	<0,03			POS	NEG	NOT	EQUAL	1
01	01-0048	51	F	1	N	Yes	NON	NA	NA	NA	NA	NA	NA	NA	NA	NEG	17			NEG	6	NEG	23			<0,03			NEG	NEG	NOT	EQUAL	1
01	01-0049	53	F	1	N	Yes	HCV	NA	NA	C	NA	NA	NA	N	NA	NEG	18			POS	5	NEG	25	HCV	POS	<0,03			POS	NEG	NOT	EQUAL	1
01	01-0050	42	M	0		Yes	NON	NA	NA	NA	NA	NA	NA	NA	NA	NEG	17			NEG	6	NEG	25			NR-0,03			NEG	NEG	NOT	EQUAL	1
01	01-0051	40	M	1		Yes	HCV_ HIV	NA	NA	C	C	NA	NA	Y	Y	NEG	18			POS	5	POS	25	HCV	NEG	<0,03			POS	POS	NOT	EQUAL	1
01	01-0052	42	F	1	N	Yes	HCV	NA	NA	C	NA	NA	NA	Y	NA	NEG	18			POS	6	NEG	24	HCV	NEG	<0,03			POS	NEG	NOT	EQUAL	1
01	01-0053	62	M	0		Yes	NON	NA	NA	NA	NA	NA	NA	NA	NA	POS	22		POS	NEG	7	NEG	33	HBV	NEG	0,18	POS	0,540	NEG	NEG	NOT	EQUAL	1
01	01-0054	30	M	0		Yes	NON	NA	NA	NA	NA	NA	NA	NA	NA	NEG	22			NEG	10	NEG	30			<0,03			NEG	NEG	NOT	EQUAL	1
01	01-0055	43	F	1	N	Yes	NON	NA	NA	NA	NA	NA	NA	NA	NA	NEG	20			NEG	6	NEG	27			<0,03			NEG	NEG	NOT	EQUAL	1
01	01-0056	47	F	1	N	Yes	HCV_ HIV	NA	NA	C	C	NA	NA	Y	Y	NEG	16			POS	5	POS	23	HCV	NEG	<0,03			POS	POS	NOT	EQUAL	1
01	01-0057	48	M	1		Yes	NON	NA	NA	NA	NA	NA	NA	NA	NA	NEG	17			NEG	7	NEG	32			<0,03			NEG	NEG	NOT	EQUAL	1
01	01-0058	51	M	1		Yes	HCV	NA	NA	C	NA	NA	NA	Y	NA	NEG	19			POS	6	NEG	27	HCV	NEG	<0,03			POS	NEG	NOT	EQUAL	1
01	01-0059	58	M	1		Yes	HCV	NA	NA	C	NA	NA	NA	Y	NA	NEG	17			POS	5	NEG	24	HCV	NEG	<0,03			POS	NEG	NOT	EQUAL	1
01	01-0060	42	M	1		Yes	HCV_ HIV	NA	NA	C	C	NA	NA	Y	Y	NEG	19			POS	8	POS	23	HCV	NEG	<0,03			POS	POS	NOT	EQUAL	1
01	01-0061	57	M	0		Yes	HCV	NA	NA	C	NA	NA	NA	Y	NA	NEG	12	1		POS	8	NEG	32	HCV	NEG	<0,03			POS	NEG	NOT	EQUAL	1
01	01-0062	54	F	1	N	Yes	NON	NA	NA	NA	NA	NA	NA	NA	NA	NEG	15			NEG	8	NEG	28			<0,03			NEG	NEG	NOT	EQUAL	1
01	01-0063	61	M	1		Yes	NON	NA	NA	NA	NA	NA	NA	NA	NA	NEG	20			NEG	14	NEG	26			<0,03			NEG	NEG	NOT	EQUAL	1
01	01-0064	24	F	1	N	Yes	NON	NA	NA	NA	NA	NA	NA	NA	NA	NEG	16			NEG	5	NEG	22			<0,03			NEG	NEG	NOT	EQUAL	1
01	01-0065	54	F	1	N	Yes	HCV	NA	NA	C	NA	NA	NA	N	NA	NEG	20			POS	10	NEG	26	HCV	NEG	<0,03			POS	NEG	NOT	EQUAL	1
01	01-0066	33	M	1		Yes	HCV	NA	NA	C	NA	NA	NA	N	NA	NEG	18			POS	6	NEG	24	HCV	NEG	<0,03			POS	NEG	NOT	EQUAL	1
01	01-0067	42	M	1		Yes	NON	NA	NA	NA	NA	NA	NA	NA	NA	NEG	16			NEG	6	NEG	22			<0,03			NEG	NEG	ACCEPT	LESS	1
01	01-0068	33	F	1	N	Yes	NON	NA	NA	NA	NA	NA	NA	NA	NA	NEG	19			NEG	7	NEG	25			<0,03			NEG	NEG	NOT	EQUAL	1
01	01-0069	47	F	0	N	Yes	NON	NA	NA	NA	NA	NA	NA	NA	NA	NEG	16			NEG	5	NEG	22			<0,03			NEG	NEG	NOT	EQUAL	1
01	01-0070	38	F	1	N	Yes	NON	NA	NA	NA	NA	NA	NA	NA	NA	NEG	18			NEG	6	NEG	23			<0,03			NEG	NEG	NOT	EQUAL	1
01	01-0071	48	M	1		Yes	NON	NA	NA	NA	NA	NA	NA	NA	NA	NEG	17			NEG	5	NEG	23			<0,03			NEG	NEG	NOT	EQUAL	1
01	01-0072	46	F	1	N	Yes	HCV	NA	NA	C	NA	NA	NA	N	NA	NEG	16			POS	6	NEG	24	HCV	POS	<0,03			POS	NEG	NOT	EQUAL	1

01	01-0073	32	M	1		Yes	NON	NA	NA	NA	NA	NA	NA	NA	NA	NEG	17		NEG	7	NEG	26			<0,03			NEG		NEG	NOT	EQUAL	1
01	01-0074	35	M	0		Yes	HBV_HCV_HIV	NA	C	C	C	NA	N	Y	Y	POS	15		POS	6	POS	21	HCV	NEG	>150			POS		POS	NOT	EQUAL	1
01	01-0075	39	F	1	N	Yes	HCV_HIV	NA	NA	C	C	NA	NA	Y	Y	NEG	16		POS	5	POS	21	HCV	NEG	<0,03			POS		POS	NOT	EQUAL	1
01	01-0076	51	M	1		Yes	HCV_HIV	NA	NA	C	C	NA	NA	Y	Y	NEG	18		POS	7	POS	26	HCV	POS	<0,03			POS		POS	NOT	EQUAL	1
01	01-0077	61	M	1		Yes	HCV_HIV	NA	NA	C	C	NA	NA	N	Y	NEG	21		POS	13	POS	25	HCV	POS	<0,03			POS		POS	NOT	EQUAL	1
01	01-0078	53	F	1	N	Yes	HCV	NA	NA	C	NA	NA	NA	N	NA	NEG	17		POS	7	NEG	24	HCV	POS	<0,03			POS		NEG	NOT	EQUAL	1
01	01-0079	48	F	1	N	Yes	HCV	NA	NA	C	NA	NA	NA	N	NA	NEG	18		POS	7	NEG	25	HCV	POS	<0,03			POS		NEG	ACCEPT	EQUAL	1
01	01-0080	37	M	0		Yes	HCV	P	NA	C	NA	N	NA	Y	NA	NEG	21		POS	5	NEG	22	HCV	POS	<0,03			POS		NEG	NOT	EQUAL	1
01	01-0081	41	M	0		Yes	HCV	NA	NA	C	NA	NA	NA	N	NA	NEG	17		POS	6	NEG	23	HCV	NEG	<0,03			POS		NEG	NOT	EQUAL	1
01	01-0082	51	M	1		Yes	HCV_HIV	NA	NA	C	C	NA	NA	N	Y	NEG	18		POS	5	POS	24	HCV	POS	<0,03			POS		POS	NOT	EQUAL	1
01	01-0083	48	M	1		Yes	HBV_HCV	P	C	C	NA	N	N	Y	NA	NEG	16		POS	5	NEG	24	HCV	NEG	<0,03			POS		NEG	NOT	EQUAL	1
01	01-0084	44	F	1	N	Yes	NON	NA	NA	NA	NA	NA	NA	NA	NA	NEG	17		NEG	5	NEG	23			<0,03			NEG		NEG	NOT	EQUAL	1
01	01-0085	39	M	0		Yes	HBV	P	C	NA	NA	N	Y	NA	NA	NEG	17	NEG	NEG	6	NEG	24	HBV	NEG	0,081	POS	0,080	NEG		NEG	NOT	EQUAL	1
01	01-0086	36	M	1		Yes	NON	NA	NA	NA	NA	NA	NA	NA	NA	NEG	18		NEG	5	NEG	33			<0,03			NEG		NEG	NOT	EQUAL	2
01	01-0087	25	M	0		Yes	NON	NA	NA	NA	NA	NA	NA	NA	NA	NEG	17		NEG	5	NEG	22			NR 0,031			NEG		NEG	NOT	EQUAL	1
01	01-0088	55	F	1	N	Yes	HBV_HIV	NA	C	NA	C	NA	Y	NA	Y	NEG	18		POS	6	POS	24	HCV	POS	<0,03			POS		POS	NOT	EQUAL	1
01	01-0089	52	M	1		Yes	HCV	NA	NA	C	NA	NA	NA	Y	NA	NEG	16		POS	5	NEG	22	HCV	POS	<0,03			POS		NEG	NOT	EQUAL	1
01	01-0090	33	M	0		Yes	HCV	NA	NA	C	NA	NA	NA	N	NA	NEG	16		POS	6	NEG	23	HCV	POS	<0,03			POS		NEG	NOT	EQUAL	1
01	01-0091	29	M	0		Yes	HCV	NA	NA	C	NA	NA	NA	N	NA	NEG	18		POS	6	NEG	26	HCV	POS	<0,03			POS		NEG	NOT	EQUAL	1
01	01-0092	38	M	1		Yes	NON	NA	NA	NA	NA	NA	NA	NA	NA	NEG	16		NEG	5	NEG	23			NR 0,03			NEG		NEG	NOT	EQUAL	1
01	01-0093	41	F	1	N	Yes	NON	NA	NA	NA	NA	NA	NA	NA	NA	NEG	17		NEG	5	NEG	23			<0,03			NEG		NEG	NOT	EQUAL	1
01	01-0094	49	M	0		Yes	HCV	NA	NA	C	NA	NA	NA	N	NA	NEG	16		POS	5	NEG	22	HCV	NEG	<0,03			POS		NEG	NOT	EQUAL	1
01	01-0095	64	F	0	N	Yes	NON	NA	NA	NA	NA	NA	NA	NA	NA	NEG	18		NEG	9	NEG	24			<0,03			NEG		NEG	NOT	EQUAL	1
01	01-0096	43	M	0		Yes	NON	NA	NA	NA	NA	NA	NA	NA	NA	NEG	17		NEG	9	NEG	27			<0,03			NEG		NEG	NOT	EQUAL	1
01	01-0097	58	M	0		Yes	NON	P	NA	NA	NA	N	NA	NA	NA	NEG	21		NEG	8	NEG	31			<0,03			NEG		NEG	NOT	EQUAL	1
01	01-0098	47	M	0		Yes	NON	NA	NA	NA	NA	NA	NA	NA	NA	NEG	21		NEG	8	NEG	35			<0,03			NEG		NEG	NOT	EQUAL	1
01	01-0099	38	F	0	N	Yes	NON	NA	NA	NA	NA	NA	NA	NA	NA	NEG	19		NEG	10	NEG	37	HCV	NEG	<0,03			POS	NEG	NEG	ACCEPT	LESS	1
01	01-0100	46	F	1	N	Yes	NON	NA	NA	NA	NA	NA	NA	NA	NA	NEG	17		NEG	5	NEG	24			<0,03			NEG		NEG	NOT	EQUAL	1
01	01-0101	20	F	0	N	Yes	NON	NA	NA	NA	NA	NA	NA	NA	NA	NEG	18		NEG	7	NEG	25			<0,03			NEG		NEG	NOT	EQUAL	1
01	01-0102	33	F	0	N	Yes	NON	NA	NA	NA	NA	NA	NA	NA	NA	NEG	18		NEG	7	NEG	24			<0,03			NEG		NEG	NOT	EQUAL	1
01	01-0103	43	F	0	N	Yes	NON	NA	NA	NA	NA	NA	NA	NA	NA	NEG	21		NEG	7	NEG	25			<0,03			NEG		NEG	NOT	EQUAL	1
01	01-0104	32	F	0	N	Yes	NON	NA	NA	NA	NA	NA	NA	NA	NA	NEG	20		NEG	9	NEG	26			<0,03			NEG		NEG	NOT	EQUAL	1
01	01-0105	46	F	0	N	Yes	NON	NA	NA	NA	NA	NA	NA	NA	NA	NEG	21		NEG	9	NEG	28			NR0,036			NEG		NEG	NOT	LESS	1
01	01-0106	46	F	0	N	Yes	NON	NA	NA	NA	NA	NA	NA	NA	NA	NEG	21		NEG	10	NEG	22			<0,03			NEG		NEG	NOT	EQUAL	1
01	01-0107	35	F	0	N	Yes	NON	NA	NA	NA	NA	NA	NA	NA	NA	NEG	22		NEG	7	NEG	29			<0,03			NEG		NEG	NOT	EQUAL	1

01	01-0108	36	M	0		Yes	NON	NA	NA	NA	NA	NA	NA	NA	NA	NEG	17		NEG	7	NEG	28			<0,03			NEG	NEG	NOT	EQUAL	1
01	01-0109	50	M	0		Yes	NON	NA	NA	NA	NA	NA	NA	NA	NA	NEG	18		NEG	8	NEG	35			<0,03			NEG	NEG	NOT	EQUAL	1
01	01-0110	45	M	0		Yes	NON	NA	NA	NA	NA	NA	NA	NA	NA	NEG	24		NEG	8	NEG	25			<0,03			NEG	NEG	NOT	EQUAL	1
01	01-0111	19	M	0		Yes	NON	NA	NA	NA	NA	NA	NA	NA	NA	NEG	22		NEG	7	NEG	27			<0,03			NEG	NEG	NOT	EQUAL	1
01	01-0112	44	F	0	N	Yes	NON	NA	NA	NA	NA	NA	NA	NA	NA	NEG	20		NEG	8	NEG	30			<0,03			NEG	NEG	NOT	EQUAL	1
01	01-0113	36	F	0	N	Yes	NON	NA	NA	NA	NA	NA	NA	NA	NA	NEG	16		NEG	7	NEG	22			<0,03			NEG	NEG	NOT	EQUAL	1
01	01-0114	34	F	0	N	Yes	NON	NA	NA	NA	NA	NA	NA	NA	NA	NEG	18		NEG	5	NEG	24			<0,03			NEG	NEG	NOT	EQUAL	1
01	01-0115	49	F	0	N	Yes	NON	NA	NA	NA	NA	NA	NA	NA	NA	NEG	14		NEG	7	NEG	23			<0,03			NEG	NEG	NOT	LESS	1
01	01-0116	28	F	0	N	Yes	NON	NA	NA	NA	NA	NA	NA	NA	NA	NEG	16		NEG	7	NEG	23			NR-0.03			NEG	NEG	NOT	LESS	1
01	01-0117	51	F	0	N	Yes	NON	NA	NA	NA	NA	NA	NA	NA	NA	NEG	14		NEG	6	NEG	22			NR-0.033			NEG	NEG	NOT	EQUAL	1
01	01-0118	61	M	0		Yes	NON	NA	NA	NA	NA	NA	NA	NA	NA	NEG	17		NEG	9	NEG	23			<0,03			NEG	NEG	NOT	LESS	1
01	01-0119	56	F	0	N	Yes	NON	NA	NA	NA	NA	NA	NA	NA	NA	NEG	19		NEG	7	NEG	26			<0,03			NEG	NEG	NOT	LESS	1
01	01-0120	27	M	0		Yes	NON	NA	NA	NA	NA	NA	NA	NA	NA	NEG	17		NEG	5	NEG	23			<0,03			NEG	NEG	NOT	EQUAL	1
01	01-0121	34	F	0	N	Yes	NON	NA	NA	NA	NA	NA	NA	NA	NA	NEG	17		NEG	6	NEG	23			<0,03			NEG	NEG	NOT	MORE	1
01	01-0122	63	M	1		Yes	NON	NA	NA	NA	NA	NA	NA	NA	NA	NEG	17		NEG	7	NEG	26			<0,03			NEG	NEG	NOT	LESS	1
01	01-0123	25	F	1	N	Yes	NON	NA	NA	NA	NA	NA	NA	NA	NA	NEG	19		NEG	9	NEG	25			<0,03			NEG	NEG	NOT	LESS	1
01	01-0124	44	F	0	N	Yes	NON	NA	NA	NA	NA	NA	NA	NA	NA	NEG	16		NEG	7	NEG	23			<0,03			NEG	NEG	NOT	MORE	1
01	01-0125	34	F	1	N	Yes	NON	NA	NA	NA	NA	NA	NA	NA	NA	NEG	15		NEG	7	NEG	26			<0,03			NEG	NEG	NOT	LESS	1
01	01-0126	40	F	0	N	Yes	NON	P	NA	NA	NA	N	NA	NA	NA	NEG	17		NEG	5	NEG	24			<0,03			NEG	NEG	NOT	MORE	1
01	01-0127	49	M	0		Yes	NON	P	NA	NA	NA	N	NA	NA	NA	NEG	17		NEG	7	NEG	23			<0,03			NEG	NEG	NOT	LESS	1
01	01-0128	25	M	0		Yes	NON	NA	NA	NA	NA	NA	NA	NA	NA	NEG	17		NEG	5	NEG	24			<0,03			NEG	NEG	NOT	LESS	1
01	01-0130	44	F	0	N	Yes	NON	NA	NA	NA	NA	NA	NA	NA	NA	NEG	18		NEG	7	NEG	26			<0,03			NEG	NEG	NOT	EQUAL	1
01	01-0131	24	M	0		Yes	NON	NA	NA	NA	NA	NA	NA	NA	NA	NEG	16		NEG	7	NEG	28			<0,03			NEG	NEG	NOT	LESS	1
01	01-0132	31	F	0	N	Yes	NON	NA	NA	NA	NA	NA	NA	NA	NA	NEG	19		NEG	7	NEG	29			<0,03			NEG	NEG	NOT	EQUAL	1
01	01-0133	30	M	0		Yes	NON	NA	NA	NA	NA	NA	NA	NA	NA	NEG	18		NEG	8	NEG	31			<0,03			NEG	NEG	NOT	LESS	1
01	01-0134	61	F	0	N	Yes	NON	P	NA	NA	NA	N	NA	NA	NA	NEG	14		NEG	9	NEG	28			<0,03			NEG	NEG	NOT	EQUAL	1
01	01-0135	41	F	0	N	Yes	NON	NA	NA	NA	NA	NA	NA	NA	NA	NEG	18		NEG	8	NEG	32			NR-0,037			NEG	NEG	NOT	EQUAL	1
01	01-0136	30	M	0		Yes	HBV	NA	C	NA	NA	NA	Y	NA	NA	NEG	18		NEG	8	NEG	26			<0,03			NEG	NEG	NOT	EQUAL	1
01	01-0137	39	M	0		Yes	NON	NA	NA	NA	NA	NA	NA	NA	NA	NEG	18		NEG	7	NEG	25			<0,03			NEG	NEG	NOT	EQUAL	1
01	01-0138	42	F	0	N	Yes	NON	NA	NA	NA	NA	NA	NA	NA	NA	NEG	25		NEG	6	NEG	28			<0,03			NEG	NEG	NOT	EQUAL	1
01	01-0139	29	F	1	N	Yes	NON	NA	NA	NA	NA	NA	NA	NA	NA	NEG	16		NEG	6	NEG	23			<0,03			NEG	NEG	NOT	EQUAL	1
01	01-0140	40	F	1	N	Yes	HIV	NA	NA	NA	C	NA	NA	NA	Y	NEG	17		NEG	8	POS	21			<0,03			NEG	POS	NOT	LESS	1
01	01-0141	58	M	1		Yes	HCV	NA	NA	C	NA	NA	NA	N	NA	NEG	17		POS	5	NEG	22	HCV	POS	<0,03			POS	NEG	NOT	LESS	1
01	01-0143	44	M	1		Yes	HCV	NA	NA	C	NA	NA	NA	Y	NA	NEG	26	NEG	POS	5	NEG	31	HCV	NEG	NR-0,052	NEG	<0,05	POS	NEG	ACCEPT	LESS	1
01	01-0144	48	M	1		Yes	NON	NA	NA	NA	NA	NA	NA	NA	NA	NEG	17		NEG	6	NEG	23			<0,03			NEG	NEG	NOT	LESS	1
01	01-0145	37	M	1		Yes	NON	NA	NA	NA	NA	NA	NA	NA	NA	NEG	17		POS	6	NEG	23	HCV	POS	<0,03			POS	NEG	ACCEPT	EQUAL	1
01	01-0146	44	M	1		Yes	HCV	NA	NA	C	NA	NA	NA	Y	NA	NEG	18		POS	6	NEG	25	HCV	NEG	<0,03			POS	NEG	ACCEPT	EQUAL	1
01	01-0147	47	M	1		Yes	HCV_ HIV	P	NA	C	C	N	NA	N	Y	NEG	17	NEG	POS	5	POS	25	HBV_H	NEG	0,22	POS	<0,05	POS	POS	NOT	LESS	1
01	01-0148	50	M	1		Yes	NON	NA	NA	NA	NA	NA	NA	NA	NA	NEG	17		NEG	5	NEG	25			NR-0,036			NEG	NEG	ACCEPT	LESS	1
01	01-0149	46	F	1	N	Yes	HIV	NA	NA	NA	C	NA	NA	NA	Y	NEG	18		POS	8	POS	29	HCV	NEG	<0,03			POS	POS	NOT	EQUAL	1
01	01-0150	48	F	1	N	Yes	HCV	NA	NA	C	NA	NA	NA	Y	NA	NEG	16		POS	5	NEG	22	HCV	NEG	<0,03			POS	NEG	ACCEPT	LESS	1
01	01-0151	57	M	0		Yes	NON	NA	NA	NA	NA	NA	NA	NA	NA	NEG	17		NEG	5	NEG	22			<0,03			NEG	NEG	NOT	EQUAL	1
01	01-0152	33	M	1		Yes	HCV	NA	NA	C	NA	NA	NA	Y	NA	NEG	19		POS	9	NEG	28	HCV	NEG	<0,03			POS	NEG	NOT	EQUAL	2

01	01-0153	47	M	1		Yes	HCV_ HIV	NA	NA	C	C	NA	NA	Y	Y	NEG	18		POS	5	POS	23		HCV	NEG	<0,03			POS		POS		ACCEPT	EQUAL	1
01	01-0154	46	M	1		Yes	NON	NA	NA	NA	NA	NA	NA	NA	NA	NEG	21		NEG	12	NEG	27				<0,03			NEG		NEG		NOT	LESS	1
01	01-0155	36	M	1		Yes	NON	NA	NA	NA	NA	NA	NA	NA	NA	NEG	19		NEG	9	NEG	31				<0,03			NEG		NEG		ACCEPT	LESS	2
01	01-0156	56	M	1		Yes	HCV	NA	NA	C	NA	NA	NA	Y	NA	NEG	19		POS	6	NEG	26		HCV	NEG	<0,03			POS		NEG		NOT	LESS	1
01	01-0157	48	M	1		Yes	HCV	NA	NA	C	NA	NA	NA	Y	NA	NEG	18	NEG	POS	7	NEG	28		HBV_H	NEG	0,14	POS	<0,05	POS		NEG		NOT	LESS	1
01	01-0158	58	M	1		Yes	HCV	NA	NA	C	NA	NA	NA	Y	NA	NEG	19		POS	8	NEG	36		HCV	NEG	<0,03			POS		NEG		NOT	LESS	1
01	01-0159	50	F	1	N	Yes	NON	NA	NA	NA	NA	NA	NA	NA	NA	NEG	29		NEG	18	NEG	27				<0,03			NEG		NEG		NOT	EQUAL	1
01	01-0160	51	M	1		Yes	HCV_ HIV	NA	NA	C	C	NA	NA	N	Y	NEG	17		POS	5	POS	23		HCV	POS	<0,03			POS		POS		NOT	LESS	1
01	01-0161	58	M	1		Yes	NON	NA	NA	NA	NA	NA	NA	NA	NA	NEG	17		NEG	5	NEG	25				<0,03			NEG		NEG		ACCEPT	EQUAL	1
01	01-0162	33	M	0		Yes	HCV_ HIV	NA	NA	C	C	NA	NA	N	Y	NEG	16		POS	6	POS	23		HCV	POS	<0,03			POS		POS		NOT	EQUAL	1
01	01-0163	42	M	1		Yes	NON	NA	NA	NA	NA	NA	NA	NA	NA	NEG	15		NEG	5	NEG	22				<0,03			NEG		NEG		NOT	EQUAL	1
01	01-0164	45	F	1	N	Yes	NON	NA	NA	NA	NA	NA	NA	NA	NA	NEG	17		NEG	5	NEG	23				<0,03			NEG		NEG		NOT	EQUAL	1
01	01-0165	30	M	1		Yes	NON	NA	NA	NA	NA	NA	NA	NA	NA	NEG	17		NEG	9	NEG	23				<0,03			NEG		NEG		NOT	EQUAL	1
01	01-0166	61	F	1	N	Yes	HCV	NA	NA	C	NA	NA	NA	N	NA	NEG	19		POS	7	NEG	27		HCV	POS	<0,03			POS		NEG		NOT	EQUAL	1
01	01-0167	41	M	1		Yes	NON	NA	NA	NA	NA	NA	NA	NA	NA	NEG	19		NEG	7	NEG	25				<0,03			NEG		NEG		NOT	LESS	1
01	01-0168	57	M	1		Yes	HBV_ HCV	NA	C	C	NA	NA	N	N	NA	NEG	17		NEG	6	NEG	23				<0,03			NEG		NEG		NOT	EQUAL	1
01	01-0169	32	M	0		Yes	NON	NA	NA	NA	NA	NA	NA	NA	NA	NEG	17		NEG	7	NEG	24				<0,03			NEG		NEG		NOT	EQUAL	1
01	01-0170	59	M	1		Yes	HBV_ HCV	NA	C	C	NA	NA	N	N	NA	NEG	18		POS	6	NEG	45	1	HCV	POS	<0,03			POS		NEG		NOT	MORE	1
01	01-0171	59	M	1		Yes	HBV_ HCV	NA	C	C	NA	NA	N	N	NA	NEG	21		NEG	11	NEG	29				<0,03			NEG		NEG		NOT	EQUAL	1
01	01-0172	60	F	1	N	Yes	HBV_ HCV	NA	C	C	NA	NA	N	N	NA	NEG	21		NEG	8	NEG	27		HCV	NEG	<0,03			POS	POS	NEG		NOT	LESS	1
01	01-0173	55	M	1		Yes	HBV	P	C	NA	NA	N	N	NA	NA	POS	22		POS	8	NEG	26		HCV	NEG	>150			POS		NEG		NOT	LESS	1
01	01-0174	49	F	1	N	Yes	HIV	NA	NA	NA	C	NA	NA	NA	Y	NEG	17		NEG	7	POS	26				<0,03			NEG		POS		NOT	MORE	1
01	01-0175	48	M	1		Yes	HIV	NA	NA	NA	C	NA	NA	NA	Y	NEG	18		POS	6	POS	27		HCV	NEG	NR-0,031			POS		POS		NOT	LESS	1
01	01-0176	47	M	1		Yes	HCV	NA	NA	C	NA	NA	NA	N	NA	NEG	25		POS	7	NEG	23		HCV	NEG	<0,03			POS		NEG		NOT	EQUAL	1
01	01-0177	33	M	0		Yes	HCV	NA	NA	C	NA	NA	NA	N	NA	NEG	23		POS	8	NEG	20		HCV	POS	<0,03			POS		NEG		NOT	LESS	1
01	01-0178	34	M	0		Yes	NON	NA	NA	NA	NA	NA	NA	NA	NA	NEG	18		POS	9	NEG	31		HCV	POS	NR-0,042			POS		NEG		NOT	EQUAL	2
01	01-0179	56	F	1	N	Yes	HCV	NA	NA	C	NA	NA	NA	N	NA	NEG	21		POS	7	NEG	28		HCV	NEG	<0,03			POS		NEG		NOT	LESS	1
01	01-0180	47	M	1		Yes	HCV	NA	NA	C	NA	NA	NA	N	NA	NEG	25		POS	13	NEG	25		HCV	NEG	<0,03			POS		NEG		NOT	EQUAL	1
01	01-0181	47	M	1		Yes	HCV	NA	NA	C	NA	NA	NA	N	NA	NEG	21		POS	9	NEG	26		HCV	NEG	<0,03			POS		NEG		NOT	LESS	1
01	01-0182	39	F	1	N	Yes	NON	NA	NA	NA	NA	NA	NA	NA	NA	NEG	18		NEG	8	NEG	30		HIV	NEG	NR-0,035			NEG		POS	NEG	NOT	LESS	1
01	01-0183	48	F	1	N	Yes	HCV_ HIV	NA	NA	C	C	NA	NA	N	Y	NEG	24		POS	18	POS	35		HCV	NEG	<0,03			POS		POS		NOT	EQUAL	1
01	01-0184	64	M	1		Yes	NON	NA	NA	NA	NA	NA	NA	NA	NA	NEG	19		NEG	8	NEG	32				<0,03			NEG		NEG		NOT	EQUAL	1

01	01-0185	43	M	1		Yes	NON	NA	NA	NA	NA	NA	NA	NA	NA	NEG	21			NEG	7		NEG	37			<0,03			NEG	NEG	NOT	EQUAL	1
01	01-0186	55	M	1		Yes	HCV	NA	NA	C	NA	NA	NA	N	NA	NEG	19			POS	7		NEG	34	HCV	NEG	<0,03			POS	NEG	NOT	EQUAL	1
01	01-0187	42	F	1	N	Yes	NON	NA	NA	NA	NA	NA	NA	NA	NA	NEG	21			NEG	8		NEG	29			<0,03			NEG	NEG	NOT	LESS	1
01	01-0188	44	F	1	N	Yes	NON	NA	NA	NA	NA	NA	NA	NA	NA	NEG	19			NEG	8		NEG	26			<0,03			NEG	NEG	NOT	LESS	1
01	01-0189	54	M	1		Yes	NON	NA	NA	NA	NA	NA	NA	NA	NA	NEG	29		NEG	NEG	7		NEG	37	HBV	NEG	0,16	POS	<0,05	NEG	NEG	NOT	MORE	1
01	01-0190	52	M	1		Yes	NON	NA	NA	NA	NA	NA	NA	NA	NA	NEG	17		NEG	NEG	7		NEG	23	HBV	NEG	0,60	NEG	<0,05	NEG	NEG	NOT	LESS	1
01	01-0191	47	M	1		Yes	NON	NA	NA	NA	NA	NA	NA	NA	NA	NEG	17			NEG	8		NEG	37			<0,03			NEG	NEG	NOT	LESS	1
01	01-0192	39	M	1		Yes	HBV	NA	C	NA	NA	NA	N	NA	NA	NEG	16			NEG	7		NEG	28			<0,03			NEG	NEG	NOT	LESS	1
01	01-0193	47	M	1		Yes	NON	NA	NA	NA	NA	NA	NA	NA	NA	NEG	20			NEG	8		NEG	34			NR-0,041			NEG	NEG	NOT	LESS	1
01	01-0194	44	M	0		Yes	HCV	NA	NA	C	NA	NA	NA	N	NA	NEG	21			POS	17		NEG	27	HCV	NEG	<0,03			POS	NEG	NOT	EQUAL	1
01	01-0195	27	M	1		Yes	NON	NA	NA	NA	NA	NA	NA	NA	NA	NEG	21			NEG	8		NEG	28			<0,03			NEG	NEG	NOT	EQUAL	1
01	01-0196	37	F	1	N	Yes	NON	NA	NA	NA	NA	NA	NA	NA	NA	NEG	19			NEG	7		NEG	33			NR-0,032			NEG	NEG	NOT	EQUAL	1
01	01-0198	52	M	1		Yes	NON	NA	NA	NA	NA	NA	NA	NA	NA	NEG	19		NEG	NEG	8		NEG	35	HBV	NEG	0,081	NEG	<0,05	NEG	NEG	NOT	EQUAL	1
01	01-0199	64	M	0		Yes	NON	NA	NA	NA	NA	NA	NA	NA	NA	NEG	29					1	NEG	25			<0,03			NEG	NEG	NOT	EQUAL	1
01	01-0200	56	M	1		Yes	NON	NA	NA	NA	NA	NA	NA	NA	NA	NEG	34	1		NEG	13		NEG	25			<0,03			NEG	NEG	NOT	EQUAL	1
01	01-0201	59	M	1		Yes	NON	NA	NA	NA	NA	NA	NA	NA	NA	NEG	15			NEG	7		NEG	20			<0,03			NEG	NEG	NOT	EQUAL	1
01	01-0202	57	M	1		Yes	NON	NA	NA	NA	NA	NA	NA	NA	NA	NEG	20			NEG	5		NEG	20			<0,03			NEG	NEG	NOT	EQUAL	1
01	01-0203	44	M	1		Yes	HCV	NA	NA	C	NA	NA	NA	N	NA	NEG	30			POS	20		NEG	20	HCV	POS	<0,03			POS	NEG	NOT	EQUAL	1
01	01-0204	60	M	1		Yes	HCV	NA	NA	C	NA	NA	NA	N	NA	NEG	24			POS	13		NEG	25	HCV	NEG	<0,03			POS	NEG	NOT	EQUAL	1
01	01-0205	60	M	1		Yes	NON	NA	NA	NA	NA	NA	NA	NA	NA	NEG	26			NEG	15		NEG	27			NR-0,033			NEG	NEG	NOT	EQUAL	1
01	01-0206	35	M	1		Yes	NON	NA	NA	NA	NA	NA	NA	NA	NA	NEG	30			NEG	20		NEG	20			<0,03			NEG	NEG	NOT	EQUAL	1
01	01-0207	46	M	0		Yes	NON	NA	NA	NA	NA	NA	NA	NA	NA	NEG	25			NEG	9		NEG	21			<0,03			NEG	NEG	NOT	LESS	1
01	01-0208	37	M	0		Yes	NON	NA	NA	NA	NA	NA	NA	NA	NA	NEG	19			NEG	19		NEG	25			<0,03			NEG	NEG	NOT	EQUAL	1
01	01-0209	52	M	1		Yes	NON	NA	NA	NA	NA	NA	NA	NA	NA	NEG	20			NEG	6		NEG	21			NR-0,035			NEG	NEG	NOT	LESS	1
01	01-0211	34	M	0		Yes	NON	NA	NA	NA	NA	NA	NA	NA	NA	NEG	31			NEG	14		NEG	30			<0,03			NEG	NEG	ACCEPT	EQUAL	1
01	01-0212	47	M	1		Yes	NON	NA	NA	NA	NA	NA	NA	NA	NA	NEG	21			NEG	11		NEG	25			NR-0,044			NEG	NEG	ACCEPT	LESS	1
01	01-0213	34	M	1		Yes	NON	NA	NA	NA	NA	NA	NA	NA	NA	NEG	15		NEG	NEG	20		NEG	21	HBV	NEG	0,071	NEG	<0,05	NEG	NEG	ACCEPT	EQUAL	1
01	01-0214	31	F	0	N	Yes	NON	NA	NA	NA	NA	NA	NA	NA	NA	NEG	24			NEG	17		NEG	30			<0,03			NEG	NEG	NOT	EQUAL	1
01	01-0215	48	M	1		Yes	NON	NA	NA	NA	NA	NA	NA	NA	NA	NEG	19			NEG	23	1	NEG	30			<0,03			NEG	NEG	NOT	EQUAL	1
01	01-0216	41	M	0		Yes	NON	NA	NA	NA	NA	NA	NA	NA	NA	NEG	31			NEG	15		NEG	32			<0,03			NEG	NEG	NOT	EQUAL	1
01	01-0217	53	M	0		Yes	NON	NA	NA	NA	NA	NA	NA	NA	NA	NEG	19			NEG	23	1	NEG	28			NR-0,044			NEG	NEG	NOT	EQUAL	1
01	01-0218	43	M	1		Yes	NON	NA	NA	NA	NA	NA	NA	NA	NA	NEG	23			NEG	16		NEG	26			NR-0,032			NEG	NEG	ACCEPT	EQUAL	1
01	01-0219	54	F	1	N	Yes	NON	NA	NA	NA	NA	NA	NA	NA	NA	NEG	17			NEG	7		NEG	26			<0,03			NEG	NEG	NOT	EQUAL	1
01	01-0220	33	M	0		Yes	NON	NA	NA	NA	NA	NA	NA	NA	NA	NEG	21			NEG	16		NEG	23			<0,03			NEG	NEG	NOT	LESS	1
01	01-0221	64	M	0		Yes	NON	NA	NA	NA	NA	NA	NA	NA	NA	NEG	24			NEG	13		NEG	20			<0,03			NEG	NEG	NOT	EQUAL	1
01	01-0222	23	M	1		Yes	NON	NA	NA	NA	NA	NA	NA	NA	NA	NEG	22			NEG	16		NEG	28			<0,03			NEG	NEG	NOT	EQUAL	1
01	01-0223	61	M	1		Yes	NON	NA	NA	NA	NA	NA	NA	NA	NA	NEG	30			NEG	15		NEG	21			<0,03			NEG	NEG	NOT	EQUAL	1
01	01-0224	48	F	0	N	Yes	NON	NA	NA	NA	NA	NA	NA	NA	NA	NEG	29			NEG	18		NEG	33			<0,03			NEG	NEG	ACCEPT	LESS	1
01	01-0225	59	M	0		Yes	NON	NA	NA	NA	NA	NA	NA	NA	NA	NEG	17			NEG	8		NEG	28			NR-0,039			NEG	NEG	NOT	EQUAL	1
01	01-0226	64	M	1		Yes	HCV	NA	NA	C	NA	NA	NA	N	NA	NEG	12	1		NEG	7		NEG	26			<0,03			NEG	NEG	NOT	MORE	1
01	01-0227	42	M	0		Yes	NON	NA	NA	NA	NA	NA	NA	NA	NA	NEG	21			NEG	8		NEG	38			<0,03			NEG	NEG	NOT	LESS	1
01	01-0228	42	M	0		Yes	NON	P	NA	NA	NA	N	NA	NA	NA	NEG	16		NEG	NEG	7		NEG	24	HBV	NEG	0,05	NEG	<0,05	NEG	NEG	NOT	LESS	1
01	01-0229	54	M	0		Yes	NON	NA	NA	NA	NA	NA	NA	NA	NA	NEG	16			NEG	19		NEG	20			<0,03			NEG	NEG	NOT	EQUAL	1
01	01-0230	40	M	0		Yes	NON	NA	NA	NA	NA	NA	NA	NA	NA	NEG	31			NEG	27	1	NEG	22			NR-0,034			NEG	NEG	ACCEPT	EQUAL	1
01	01-0231	49	M	0		Yes	NON	NA	NA	NA	NA	NA	NA	NA	NA	NEG	19			NEG	13		NEG	30			<0,03			NEG	NEG	NOT	MORE	1

01	01-0232	20	M	0		Yes	NON	NA	NA	NA	NA	NA	NA	NA	NA	NEG	17			NEG	14		NEG	22				<0,03			NEG	NEG	NOT	EQUAL	1
01	01-0233	30	M	0		Yes	NON	NA	NA	NA	NA	NA	NA	NA	NA	NEG	24		NEG	NEG	18		NEG	35		HBV	NEG	0,09	NEG	<0,05	NEG	NEG	ACCEPT	EQUAL	1
01	01-0234	50	M	1		Yes	NON	NA	NA	NA	NA	NA	NA	NA	NA	NEG	27			NEG	25	1	NEG	29				<0,03			NEG	NEG	NOT	EQUAL	1
01	01-0236	63	F	0	N	Yes	NON	NA	NA	NA	NA	NA	NA	NA	NA	NEG	20			NEG	12		NEG	26				<0,03			NEG	NEG	NOT	EQUAL	1
01	01-0237		F	1	N	Yes	NON	NA	NA	NA	NA	NA	NA	NA	NA	NEG	28			NEG	17		NEG	24				NR-0,038			NEG	NEG	NOT	EQUAL	1
01	01-0238	45	F	1	N	Yes	NON	NA	NA	NA	NA	NA	NA	NA	NA	NEG	24			NEG	19		NEG	28				NR-0,037			NEG	NEG	NOT	EQUAL	1
01	01-0239	55	M	1		Yes	NON	NA	NA	NA	NA	NA	NA	NA	NA	NEG	23			NEG	15		NEG	21				<0,03			NEG	NEG	NOT	LESS	1
01	01-0240	48	F	1	N	Yes	NON	NA	NA	NA	NA	NA	NA	NA	NA	NEG	25			NEG	20		NEG	20				<0,03			NEG	NEG	NOT	LESS	1
01	01-0241	47	F	0	N	Yes	NON	NA	NA	NA	NA	NA	NA	NA	NA	NEG	22		NEG	NEG	6		NEG	23		HBV	NEG	0,09	NEG	<0,05	NEG	NEG	NOT	LESS	1
01	01-0242	73	M	1		Yes	NON	NA	NA	NA	NA	NA	NA	NA	NA	NEG	31		NEG	NEG	20		NEG	29		HBV	NEG	1	POS	<0,05	NEG	NEG	NOT	LESS	1
01	01-0243	54	M	1		Yes	NON	NA	NA	NA	NA	NA	NA	NA	NA	NEG	24		NEG	NEG	9		NEG	25		HBV	NEG	0,22	POS	<0,05	NEG	NEG	ACCEPT	LESS	1
01	01-0244	50	M	0		Yes	NON	NA	NA	NA	NA	NA	NA	NA	NA	NEG	22			NEG	16		NEG	37				<0,03			NEG	NEG	ACCEPT	LESS	1
01	01-0245	48	M	0		Yes	NON	NA	NA	NA	NA	NA	NA	NA	NA	NEG	26			NEG	18		NEG	31				<0,03			NEG	NEG	NOT	EQUAL	1
01	01-0246	54	M	1		Yes	HCV	NA	NA	C	NA	NA	NA	N	NA	NEG	19			POS	9		NEG	26		HCV	NEG	<0,03			POS	NEG	NOT	LESS	1
01	01-0247	45	M	0		Yes	NON	NA	NA	NA	NA	NA	NA	NA	NA	NEG	23			NEG	8		NEG	23				<0,03			NEG	NEG	ACCEPT	LESS	1
01	01-0248	50	F	1	N	Yes	HCV	NA	NA	C	NA	NA	NA	N	NA	NEG	26			POS	6		NEG	32		HCV	NEG	NR-0,032			POS	NEG	NOT	LESS	1
01	01-0249	29	F	1	N	Yes	NON	NA	NA	NA	NA	NA	NA	NA	NA	NEG	28			NEG	9		NEG	35				<0,03			NEG	NEG	NOT	EQUAL	1
01	01-0250	52	M	1		Yes	HCV	NA	NA	C	NA	NA	NA	N	NA	NEG	21			POS	10		NEG	21		HCV	NEG	<0,03			POS	NEG	NOT	LESS	1
01	01-0251	42	F	0	N	Yes	NON	NA	NA	NA	NA	NA	NA	NA	NA	NEG	17		NEG	POS	6		NEG	27		HCV	NEG	0,066	NEG	<0,05	POS	NEG	NOT	EQUAL	1
01	01-0252	52	F	0	N	Yes	NON	NA	NA	NA	NA	NA	NA	NA	NA	NEG	19			NEG	9		NEG	24				<0,03			NEG	NEG	NOT	LESS	1
01	01-0253	52	M	0		Yes	HCV	NA	NA	C	NA	NA	NA	N	NA	NEG	23			POS	6		NEG	24		HCV	NEG	<0,03			POS	NEG	NOT	EQUAL	1
01	01-0254	24	F	0	N	Yes	NON	NA	NA	NA	NA	NA	NA	NA	NA	NEG	23			NEG	7		NEG	34				<0,03			NEG	NEG	NOT	LESS	1
01	01-0255	41	M	0		Yes	NON	NA	NA	NA	NA	NA	NA	NA	NA	NEG	28			NEG	11		NEG	38				<0,03			NEG	NEG	NOT	LESS	1
01	01-0256	35	F	0	N	Yes	NON	NA	NA	NA	NA	NA	NA	NA	NA	NEG	21			NEG	7		NEG	27				<0,03			NEG	NEG	NOT	LESS	1
01	01-0257	38	M	0		Yes	NON	NA	NA	NA	NA	NA	NA	NA	NA	NEG	22			NEG	6		NEG	31				NR-0,044			NEG	NEG	NOT	LESS	1
01	01-0258	35	F	0	N	Yes	NON	NA	NA	NA	NA	NA	NA	NA	NA	NEG	21			NEG	9		NEG	27				<0,03			NEG	NEG	ACCEPT	EQUAL	1
01	01-0259	54	M	1		Yes	HCV	NA	NA	C	NA	NA	NA	N	NA	NEG	22			POS	7		NEG	22		HCV	NEG	NR-0,03			POS	NEG	ACCEPT	MORE	1
01	01-0260	50	M	0		Yes	NON	NA	NA	NA	NA	NA	NA	NA	NA	NEG	28			NEG	7		NEG	35				<0,03			NEG	NEG	NOT	LESS	1
01	01-0261	45	F	0	N	Yes	NON	NA	NA	NA	NA	NA	NA	NA	NA	NEG	24			NEG	7		NEG	32				<0,03			NEG	NEG	ACCEPT	EQUAL	1
01	01-0262	31	F	0	N	Yes	NON	P	NA	NA	NA	N	NA	NA	NA	NEG	21			NEG	11		INV	85	1			<0,03			NEG	NEG	NOT	LESS	1
01	01-0263	37	M	0		Yes	NON	P	NA	NA	NA	N	NA	NA	NA	NEG	18			NEG	9		NEG	35				NR-0,031			NEG	NEG	ACCEPT	LESS	1
01	01-0264	32	F	0	N	Yes	NON	NA	NA	NA	NA	NA	NA	NA	NA	NEG	35	1		NEG	7		NEG	43	1			<0,03			NEG	NEG	NOT	LESS	1
01	01-0265	32	M	0		Yes	NON	NA	NA	NA	NA	NA	NA	NA	NA	NEG	31			NEG	8		NEG	47	1			NR-0,035			NEG	NEG	ACCEPT	MORE	1
01	01-0266	51	F	0	N	Yes	NON	NA	NA	NA	NA	NA	NA	NA	NA	NEG	19			NEG	8		NEG	25				<0,03			NEG	NEG	ACCEPT	MORE	1
01	01-0267	76	M	1		Yes	NON	NA	NA	NA	NA	NA	NA	NA	NA	NEG	20			NEG	15		NEG	30				<0,03			NEG	NEG	ACCEPT	LESS	1
01	01-0268	37	M	0		Yes	NON	P	NA	NA	NA	N	NA	NA	NA	NEG	20			NEG	8		NEG	27				<0,03			NEG	NEG	ACCEPT	MORE	1
01	01-0269	43	M	0		Yes	NON	NA	NA	NA	NA	NA	NA	NA	NA	NEG	23			NEG	8		NEG	31				<0,03			NEG	NEG	NOT	LESS	1
01	01-0270	41	F	0	N	Yes	NON	NA	NA	NA	NA	NA	NA	NA	NA	NEG	27			NEG	9		NEG	35				NR-0,031			NEG	NEG	ACCEPT	MORE	1
01	01-0271	39	F	0	N	Yes	NON	NA	NA	NA	NA	NA	NA	NA	NA	NEG	20			NEG	7		NEG	32				NR-0,03			NEG	NEG	ACCEPT	MORE	1
01	01-0272	40	M	0		Yes	NON	NA	NA	NA	NA	NA	NA	NA	NA	NEG	24			POS	7		NEG	37		HCV	POS	<0,03			POS	NEG	NOT	LESS	1
01	01-0273	37	F	0	N	Yes	NON	NA	NA	NA	NA	NA	NA	NA	NA	NEG	31			NEG	9		NEG	42	1			<0,03			NEG	NEG	ACCEPT	MORE	1
01	01-0274	25	F	0	N	Yes	NON	NA	NA	NA	NA	NA	NA	NA	NA	NEG	22			NEG	8		NEG	34				<0,03			NEG	NEG	NOT	EQUAL	1
01	01-0275	32	M	0		Yes	NON	NA	NA	NA	NA	NA	NA	NA	NA	NEG	18			NEG	8		NEG	33				<0,03			NEG	NEG	NOT	LESS	1
01	01-0276	36	F	0	N	Yes	NON	NA	NA	NA	NA	NA	NA	NA	NA	NEG	24		NEG	NEG	9		NEG	34		HBV	NEG	0,061	NEG	<0,05	NEG	NEG	NOT	LESS	1
01	01-0277	29	M	0		Yes	NON	NA	NA	NA	NA	NA	NA	NA	NA	NEG	24			NEG	7		NEG	32				NR-0,044			NEG	NEG	ACCEPT	EQUAL	1

01	01-0278	30	F	0	N	Yes	NON	NA	NA	NA	NA	NA	NA	NA	NA	NEG	26		NEG	7		NEG	35				<0,03			NEG	NEG	NOT	LESS	1
01	01-0279	41	F	0	N	Yes	NON	NA	NA	NA	NA	NA	NA	NA	NA	NEG	25		NEG	7		NEG	32				NR-0,034			NEG	NEG	NOT	EQUAL	1
01	01-0280	43	M	0		Yes	NON	NA	NA	NA	NA	NA	NA	NA	NA	NEG	20		NEG	8		NEG	37				NR-0,036			NEG	NEG	NOT	EQUAL	1
01	01-0281	55	F	0	N	Yes	NON	NA	NA	NA	NA	NA	NA	NA	NA	NEG	25		NEG	20		NEG	31				<0,03			NEG	NEG	ACCEPT	MORE	1
01	01-0282	21	F	0	N	Yes	NON	NA	NA	NA	NA	NA	NA	NA	NA	NEG	24		NEG	7		NEG	37				<0,03			NEG	NEG	NOT	LESS	1
01	01-0283	57	F	0	N	Yes	NON	NA	NA	NA	NA	NA	NA	NA	NA	NEG	22		NEG	9		NEG	32				<0,03			NEG	NEG	NOT	EQUAL	1
01	01-0284	25	F	0	N	Yes	NON	NA	NA	NA	NA	NA	NA	NA	NA	NEG	17		NEG	8		NEG	35				<0,03			NEG	NEG	NOT	LESS	1
01	01-0285	42	F	0	N	Yes	NON	NA	NA	NA	NA	NA	NA	NA	NA	NEG	27		NEG	8		NEG	36				NR-0,033			NEG	NEG	ACCEPT	EQUAL	1
01	01-0286	41	M	0		Yes	NON	NA	NA	NA	NA	NA	NA	NA	NA	NEG	21		NEG	11		NEG	22				<0,03			NEG	NEG	ACCEPT	LESS	1
01	01-0287	48	M	1		Yes	HCV_ HIV	NA	NA	C	C	NA	NA	N	Y	NEG	31		POS	16		POS	34		HCV	POS	<0,03			POS	POS	ACCEPT	LESS	1
01	01-0288	56	M	0		Yes	HCV	NA	NA	C	NA	NA	NA	N	NA	NEG	27	NEG	POS	11		NEG	37		HCV	NEG	0,066	NEG	<0,05	POS	NEG	NOT	EQUAL	1
01	01-0289	58	F	0	N	Yes	NON	NA	NA	NA	NA	NA	NA	NA	NA	NEG	24		POS	11		NEG	31		HCV	NEG	<0,03			POS	NEG	NOT	LESS	1
01	01-0290	60	M	0		Yes	NON	NA	NA	NA	NA	NA	NA	NA	NA	NEG	23	NEG	NEG	7		NEG	38		HBV	NEG	0,18	NEG	<0,05	NEG	NEG	NOT	LESS	1
01	01-0291	50	M	0		Yes	HCV	NA	NA	C	NA	NA	NA	N	NA	NEG	23		POS	7		NEG	23		HCV	POS	<0,03			POS	NEG	NOT	EQUAL	1
01	01-0292	59	M	1		Yes	HBV_ HCV	NA	C	C	NA	NA	N	N	NA	NEG	17		POS	8		NEG	37		HCV	POS	<0,03			POS	NEG	NOT	EQUAL	1
01	01-0293	66	M	1		Yes	NON	NA	NA	NA	NA	NA	NA	NA	NA	NEG	21	NEG	NEG	11		NEG	21		HBV	NEG	0,15	POS	<0,05	NEG	NEG	ACCEPT	LESS	1
01	01-0294	57	M	1		Yes	NON	NA	NA	NA	NA	NA	NA	NA	NA	NEG	24		POS	12		POS	36		HCV	NEG	<0,03			POS	POS	NOT	EQUAL	1
01	01-0295	36	M	0		Yes	NON	NA	NA	NA	NA	NA	NA	NA	NA	NEG	24		NEG	9		NEG	32				<0,03			NEG	NEG	NOT	LESS	1
01	01-0296	41	F	1	N	Yes	NON	NA	NA	NA	NA	NA	NA	NA	NA	NEG	19		NEG	18		NEG	24				<0,03			NEG	NEG	NOT	EQUAL	1
01	01-0297	44	F	1	N	Yes	NON	NA	NA	NA	NA	NA	NA	NA	NA	NEG	16		NEG	6		NEG	26				<0,03			NEG	NEG	NOT	EQUAL	1
01	01-0298	39	F	1	N	Yes	HCV	NA	NA	C	NA	NA	NA	N	NA	NEG	15		POS	9		NEG	21		HCV	POS	<0,03			POS	NEG	NOT	EQUAL	1
01	01-0299		F	1	N	Yes	NON	NA	NA	NA	NA	NA	NA	NA	NA	NEG	26		NEG	20		NEG	31				<0,03			NEG	NEG	ACCEPT	MORE	1
01	01-0300	26	M	0		Yes	NON	NA	NA	NA	NA	NA	NA	NA	NA	NEG	21		NEG	10		NEG	21				<0,03			NEG	NEG	NOT	LESS	1
01	01-0301	29	M	0		Yes	NON	NA	NA	NA	NA	NA	NA	NA	NA	NEG	24		NEG	21		NEG	28				<0,03			NEG	NEG	NOT	EQUAL	1
01	01-0302	57	F	0	N	Yes	NON	NA	NA	NA	NA	NA	NA	NA	NA	NEG	26		NEG	8		NEG	33				<0,03			NEG	NEG	NOT	LESS	1
01	01-0303	34	M	0		Yes	NON	NA	NA	NA	NA	NA	NA	NA	NA	NEG	25		NEG	24	1	NEG	25				<0,03			NEG	NEG	ACCEPT	MORE	1
01	01-0304	29	M	1		Yes	NON	NA	NA	NA	NA	NA	NA	NA	NA	NEG	25		NEG	9		NEG	39				<0,03			NEG	NEG	NOT	LESS	1
01	01-0305	48	F	1	N	Yes	NON	NA	NA	NA	NA	NA	NA	NA	NA	NEG	26		NEG	26	1	NEG	32				<0,03			NEG	NEG	ACCEPT	LESS	1
01	01-0306	46	M	0		Yes	NON	NA	NA	NA	NA	NA	NA	NA	NA	NEG	18		NEG	18		NEG	18	1			<0,03			NEG	NEG	ACCEPT	MORE	1
01	01-0307	31	F	1	N	Yes	NON	NA	NA	NA	NA	NA	NA	NA	NA	NEG	23		NEG	15		NEG	36				<0,03			NEG	NEG	NOT	LESS	1

**Variables**	**Description**
site	Site ID
subjID	Subject ID
age	Age
gender	Gender
spain	Origin - Spain
pregyn	If Female, pregnant?
eligible	Eligibility

diag	Has subject been diagnosed with HBV, HCV, or HIV?
Hep_A	Infectious Disease - Hepatitis A
Hep_B	Infectious Disease - Hepatitis B
Hep_C	Infectious Disease - Hepatitis C
HIV	Infectious Disease - HIV / AIDS
Hep_A_M	Is subject taking medication for Hepatitis A?
Hep_B_M	Is subject taking medication for Hepatitis B?
Hep_C_M	Is subject taking medication for Hepatitis C?
HIV_M	Is the subject taking medication for HIV?
HBV_F	Fingerstick - Result for HBV test
td_HBV	Time difference between read and inoculation for HBV test
HBV_OTW	Indicator: out of 1 min outside the time window 15-30 min for HBV test
HBV_S1	Serum, 1st Reading - Result for HBV test
HBV_S2	Serum, 2nd Reading - Result for HBV test
HCV_F	Fingerstick - Result for HCV test
td_HCV	Time difference between read and inoculation for HCV test
HCV_OTW	Indicator: out of 1 min outside the time window 5-20 min for HCV test
HIV_F	Fingerstick - Result for HIV test
td_HIV	Time difference between read and inoculation for HIV test
HIV_OTW	Indicator: out of 1 min outside the time window 20-40 min for HIV test
pcrname	PCR - Which test initiated PCR
PCR	PCR - Results
liais_hbs	LIAISON CL MUREX HBsAg Quant Result, IU/mL
neutryn	LIAISON CL MUREX HBsAg Quant Result, Neutralized or Not
antihbc	Anti-HBc Result
abbarch	Abbott Architect Result, IU/mL
liais_hcv	LIAISON CL MUREX HCV Antibody Anti-HCV Result
innolia	INNO-LIA Result
liais_hiv	LIAISON XL 4th Generation HIV Test Result
immblot	Immunoblot Result
painfs	How painful was the fingerstick?
painfsven	How painful was the fingerstick compared to the venipuncture?
manyfs	How many fingersticks were required?
